# Supplementary material for: Activator-blocker model of transcriptional regulation by pioneer-like factors
Source: Nat Commun. 2023 Sep 14;14:5677. doi: 10.1038/s41467-023-41507-z (PMC10502082; doi:10.1038/s41467-023-41507-z)
Supplement: Supplementary file 3 — Reporting Summary [file 41467_2023_41507_MOESM3_ESM.pdf]

nature portfolio

Corresponding author(s): Onichtchouk, Timmer

Last updated by author(s): Aug 4, 2023

Reporting Summary

Nature Portfolio wishes to improve the reproducibility of the work that we publish. This form provides structure for consistency and transparency in reporting. For further information on Nature Portfolio policies, see our Editorial Policies and the Editorial Policy Checklist.

Statistics

For all statistical analyses, confirm that the following items are present in the figure legend, table legend, main text, or Methods section.

n/a	Confirmed	
☐	☒	The exact sample size (*n*) for each experimental group/condition, given as a discrete number and unit of measurement
☐	☒	A statement on whether measurements were taken from distinct samples or whether the same sample was measured repeatedly
☐	☒	The statistical test(s) used AND whether they are one- or two-sided<br>*Only common tests should be described solely by name; describe more complex techniques in the Methods section.*
☐	☒	A description of all covariates tested
☐	☒	A description of any assumptions or corrections, such as tests of normality and adjustment for multiple comparisons
☐	☒	A full description of the statistical parameters including central tendency (e.g. means) or other basic estimates (e.g. regression coefficient) AND variation (e.g. standard deviation) or associated estimates of uncertainty (e.g. confidence intervals)
☐	☒	For null hypothesis testing, the test statistic (e.g. *F*, *t*, *r*) with confidence intervals, effect sizes, degrees of freedom and *P* value noted<br>*Give P values as exact values whenever suitable.*
☒	☐	For Bayesian analysis, information on the choice of priors and Markov chain Monte Carlo settings
☒	☐	For hierarchical and complex designs, identification of the appropriate level for tests and full reporting of outcomes
☐	☒	Estimates of effect sizes (e.g. Cohen's *d*, Pearson's *r*), indicating how they were calculated

Our web collection on statistics for biologists contains articles on many of the points above.

Software and code

Policy information about availability of computer code

Data collection

no software was used

Data analysis

Hermes Jacques, Rosenblatt Marcus, Activator-blocker model of transcriptional regulation by pioneer-like factors, Github vandensich/zebrafish-minimodels, 10.5281/zenodo.8211306, 2023
Bed Tools Quinlan and Hall,2010 BED Tools in usegalaxy.eu
Bowtie2 Langmead and Salzberg, 2012 Bowtie2 in usegalaxy.eu
DeepTools2 Ramirez et al., 2016 deepTools in usegalaxy.eu
DESeq2 Love et al., 2014 DESeq2 in usegalaxy.eu
FeatureCounts Liao et al., 2014 featureCounts in usegalaxy.eu
Galaxy server Afgan et al., 2018 https://usegalaxy.eu/
GREAT: Genomic Regions Enrichment of Annotations Tool, version 3.0.0 Hiller et al., 2013 http://great.stanford.edu/great/public-3.0.0/html/
In-vitro nucleosome prediction program Kaplan et al., 2009 and https://github.com/bgruening/galaxytools Nucleosome Predictions in usegalaxy.eu
k-means clustering algorithm Ramirez et al., 2016 Available option in plotheatmap in deepTools2 in usegalaxy.eu
MACS2 Ferg et al., 2007 MACS2 callpeak in usegalaxy.eu
RNA Star Dobin et al., 2013 RNA Star in usegalaxy.eu
RNA-sense https://bioconductor.org/packages/release/bioc/html/RNAsense.html
geecee utility to calculate fractional GC content of nucleic acid sequences in usegalaxy.eu
R packages

ggplot2 :

H. Wickham. ggplot2: Elegant Graphics for Data Analysis. Springer-Verlag New York, 2016.
dplyr:
Hadley Wickham, Romain FranÁois, Lionel Henry and Kirill M¸ller (2020). dplyr: A Grammar of Data Manipulation. R package version 1.0.2. https://CRAN.R-project.org/package=dplyr
eulerr:
Larsson J (2020). _eulerr: Area-Proportional Euler and Venn Diagrams with Ellipses_. R package version 6.1.0, <URL: https://cran.r-project.org/package=eulerr>.
NBPSeq:
Yanming Di, Daniel W Schafer, with contributions from Jason S Cumbie and Jeff H Chang. (2014). NBPSeq: Negative Binomial Models for RNA-Sequencing Data. R package version 0.3.0. https://CRAN.R-project.org/package=NBPSeq
qvalue:
John D. Storey, Andrew J. Bass, Alan Dabney and David Robinson (2019). qvalue: Q-value estimation for false discovery rate control. R package version 2.16.0. http://github.com/jdstorey/qvalue
SummarizedExperiment:
Martin Morgan, Valerie Obenchain, Jim Hester and HervÈ PagËs (2019). SummarizedExperiment: SummarizedExperiment container. R package version 1.14.1.
reshape2:
Hadley Wickham (2007). Reshaping Data with the reshape Package. Journal of Statistical Software, 21(12), 1-20. URL http://www.jstatsoft.org/v21/i12/.
tidyverse:
Wickham et al., (2019). Welcome to the tidyverse. Journal of Open Source Software, 4(43), 1686, https://doi.org/10.21105/joss.01686
matrixStats:
Henrik Bengtsson (2020). matrixStats: Functions that Apply to Rows and Columns of Matrices (and to Vectors). R package version 0.57.0. https://CRAN.R-project.org/package=matrixStats
hrbrthemes:
Bob Rudis (2020). hrbrthemes: Additional Themes, Theme Components and Utilities for 'ggplot2'. R package version 0.8.0. https://CRAN.R-project.org/package=hrbrthemes
viridis:
Simon Garnier (2018). viridis: Default Color Maps from 'matplotlib'. R package version 0.5.1. https://CRAN.R-project.org/package=viridis
parallel:
R Core Team (2019). R: A language and environment for statistical computing. R Foundation for Statistical Computing, Vienna, Austria. URL https://www.R-project.org/.
stats:
R Core Team (2019). R: A language and environment for statistical computing. R Foundation for Statistical Computing, Vienna, Austria. URL https://www.R-project.org/.
forcats:
Hadley Wickham (2020). forcats: Tools for Working with Categorical Variables (Factors). R package version 0.5.0. https://CRAN.R-project.org/package=forcats

For manuscripts utilizing custom algorithms or software that are central to the research but not yet described in published literature, software must be made available to editors and reviewers. We strongly encourage code deposition in a community repository (e.g. GitHub). See the Nature Portfolio guidelines for submitting code & software for further information.

Data

Policy information about availability of data

All manuscripts must include a data availability statement. This statement should provide the following information, where applicable:

- Accession codes, unique identifiers, or web links for publicly available datasets
- A description of any restrictions on data availability
- For clinical datasets or third party data, please ensure that the statement adheres to our policy

• Raw and processed RNA-seq, ChIP-seq and ATAC-seq data generated in this study have been deposited in GEO and are publicly available as of the date of publication. Accession numbers are GEO: GSE162415, GEO: GSE143439, and GEO: GSE215956

Research involving human participants, their data, or biological material

Policy information about studies with human participants or human data. See also policy information about sex, gender (identity/presentation), and sexual orientation and race, ethnicity and racism.

Reporting on sex and gender	N/A
Reporting on race, ethnicity, or other socially relevant groupings	N/A
Population characteristics	N/A
Recruitment	N/A

Ethics oversight | N/A

Note that full information on the approval of the study protocol must also be provided in the manuscript.

Field-specific reporting

Please select the one below that is the best fit for your research. If you are not sure, read the appropriate sections before making your selection.

☒ Life sciences ☐ Behavioural & social sciences ☐ Ecological, evolutionary & environmental sciences

For a reference copy of the document with all sections, see nature.com/documents/nr-reporting-summary-flat.pdf

Life sciences study design

All studies must disclose on these points even when the disclosure is negative.

Sample size

Sample sizes for RNA-seq, ChIP-seq and ATAC-seq were selected based on the common practice in the field: for RNA-seq, 60 embryos per batch (Gao, M. et al., Nat Commun 13, 788, doi:10.1038/s41467-022-28434-1 (2022); for ATAC-seq 30 embryos per batch (i.e. Palfy, M., Schulze, G., Valen, E. & Vastenhouw, N. L. , PLoS Genet 16, e1008546, doi:10.1371/journal.pgen.1008546 (2020); for ChIP-seq more than 1000 embryos were required to get enough material for the library preparation (ao, M. et al., Nat Commun 13, 788, doi:10.1038/s41467-022-28434-1 (2022), Miao, L. et al., Mol Cell 82, 986-1002 e1009, doi:10.1016/j.molcel.2022.01.024 (2022). Apart of the experiments listed here, no experiments in the zebrafish embryos were performed in the manuscript.

Data exclusions

There were no data exclusions

Replication

ATAC-seq was performed in 2-7 replicates, ChIP-seq was single replicate, RNA-seq in 2-3 replicates per genotype/time point, all the attempts of the replication were successful. Other than that, EMSA (electromobility shift assays) in-vitro experiments were performed in 2-3 replicates, all the attempts of the replication were succsessful.

Randomization

The samples were allocated to the experimental groups by genotype

Blinding

Blinding was not relevant for the study, because the genotype of the mutant zebrafish embryos can be easily recognized from their phenotypic appearance.

Reporting for specific materials, systems and methods

We require information from authors about some types of materials, experimental systems and methods used in many studies. Here, indicate whether each material, system or method listed is relevant to your study. If you are not sure if a list item applies to your research, read the appropriate section before selecting a response.

Materials & experimental systems

n/a	Involved in the study	
☐	☒	Antibodies
☒	☐	Eukaryotic cell lines
☒	☐	Palaeontology and archaeology
☐	☒	Animals and other organisms
☒	☐	Clinical data
☒	☐	Dual use research of concern
☒	☐	Plants

Methods

n/a	Involved in the study	
☐	☒	ChIP-seq
☒	☐	Flow cytometry
☒	☐	MRI-based neuroimaging

Antibodies

Antibodies used

Anti-FLAG clone M2 mouse monoclonal (Sigma F3165, lot number SLCJ3741, stock 10mM, was used in final concentration 1 mM for EMSA), anti-HA High Affinity rat monoclonal (Roche/Sigma #1186743001, Rat anti-HA High-affinity monoclonal, Clone 3F10, Lot 1564900, stock 100 µg/ml, was used in final concentration 10µg/ml for EMSA), anti-H3K27ac rabbit polyclonal (Abcam ab4729, lot number GR3357415-2, stock concentration 1 mg/ml, was used for ChIP-seq in the dilution 1/100, final concentration 10 ng/µl)

Validation

Anti- Flag is an antibody for synthetic FLAG peptide, was validated for the use with Pou5f3-FLAG in zebrafish by Leichsenring, M., Maes, J., Mossner, R., Driever, W. & Onichtchouk, D. , Science 341, 1005-1009, doi:10.1126/science.1242527 (2013). Anti-HA is an antibody for the synthetic Hemagglutinine peptide, was validated for the use with MC4R receptor- HA fusion in mouse by Eugene Nyamugenda, Haven Griffin, Susan Russell, Kimberly A. Cooney, Nicholas S. Kowalczyk, Ishrar Islam, Kevin D. Phelan, Giulia Baldini, , iScience,Volume 23, Issue 5,2020,101114,ISSN 2589-0042, https://doi.org/10.1016/j.isci.2020.101114. (https://www.sciencedirect.com/science/article/pii/S2589004220302996) https://doi.org/10.1016/j.isci.2020.101114. anti-H3K27ac rabbit polyclonal antibody was validated in zebrafish by Gao, M. et al., Nat Commun 13, 788, doi:10.1038/s41467-022-28434-1 (2022).

Animals and other research organisms

Policy information about studies involving animals; ARRIVE guidelines recommended for reporting animal research, and Sex and Gender in Research

Laboratory animals	Danio rerio (zebrafish), males and females 0-2 years old, strains: AB/TL, MZspg, MZsox19b, MZnanog, MZps, MZsn, MZpn, MZtriple
Wild animals	no wild animals were used
Reporting on sex	Fish were used for breeding only, 50 to 50 males and females
Field-collected samples	no field samples were collected
Ethics oversight	All experiments were performed in accordance with German Animal Protection Law (TierSchG) and European Convention on the Protection of Vertebrate Animals Used for Experimental and Other Scientific Purposes (Strasburg, 1986). The generation of double mutants was approved by the Ethics Committee for Animal Research of the Koltzov Institute of Developmental Biology RAS, protocol 26 from 14.02.2019

Note that full information on the approval of the study protocol must also be provided in the manuscript.

Plants

Seed stocks	N/A
Novel plant genotypes	N/A
Authentication	N/A

ChIP-seq

Data deposition

☒ Confirm that both raw and final processed data have been deposited in a public database such as GEO.

☒ Confirm that you have deposited or provided access to graph files (e.g. BED files) for the called peaks.

Data access links *May remain private before publication.*	GEO: GSE143439
Files in database submission	GSM4259504 MZnanog_H3K27ac_rep1_ChIP-seq<br>GSM4259505 MZnanog_input_for_H3K27ac
Genome browser session (e.g. UCSC)	https://genome.ucsc.edu/s/onichtchouk/danRer11_example1

Methodology

Replicates	single replicate was used due to the limited amount of material
Sequencing depth	The ChIP-seq library was sequenced at 70 mln paired end 150bp reads the input library were sequenced to 30 mln reads. Sequencing was performed by the Novogene company (China).
Antibodies	Anti-Histone H3 (acetyl K27) rabbit, 1/100 dilution Abcam plc., Cambridge, UK ab 4729
Peak calling parameters	the peaks were called with MACS2 (Feng, J., Liu, T., Qin, B., Zhang, Y. & Liu, X. S. Identifying ChIP-seq enrichment using MACS. Nat Protoc 7, 1728-1740, doi:10.1038/nprot.2012.101 (2012)), input files were pooled and used as a background. The following parameters were used:<br>Effective Genome Size=1370000000, --mfold= 5 to 50, --bw=300, --qvalue=0.05
Data quality	Quality control was performed with FastQC (Andrews, S. (n.d.). FastQC A Quality Control tool for High Throughput Sequence Data. Retrieved from http://www.bioinformatics.babraham.ac.uk/projects/fastqc/) implemented in usegalaxy. eu. The numbers of peaks at FDR 5% and above 5 fold enrichment was more than 100 000 for each ChIP sample
Software	Data processing was done in usegalaxy.eu public server (Afgan, E. et al. The Galaxy platform for accessible, reproducible and collaborative biomedical analyses: 2016 update. Nucleic Acids Res 44, W3-W10, doi:10.1093/nar/gkw343 (2016)), using Bed Tools (Quinlan, A. R. & Hall, I. M. BEDTools: a flexible suite of utilities for comparing genomic features. Bioinformatics 26, 841-842, doi:10.1093/bioinformatics/btq033 (2010)),<br>Bowtie2 (Langmead, B. & Salzberg, S. L. Fast gapped-read alignment with Bowtie 2. Nat Methods 9, 357-359, doi:10.1038/nmeth.1923 (2012)), DeepTools2 (Ramirez, F. et al. deepTools2: a next generation web server for deep-sequencing data analysis.

Nucleic Acids Res 44, W160-165, doi:10.1093/nar/gkw257 (2016)) and MACS2 (Feng, J., Liu, T., Qin, B., Zhang, Y. & Liu, X. S. Identifying ChIP-seq enrichment using MACS. Nat Protoc 7, 1728-1740, doi:10.1038/nprot.2012.101 (2012)).
