# Supplementary figures and images for: Activator-blocker model of transcriptional regulation by pioneer-like factors
Source: Nat Commun. 2023 Sep 14;14:5677. doi: 10.1038/s41467-023-41507-z (PMC10502082; doi:10.1038/s41467-023-41507-z)

**a** 2.P+N-

oligo 3 (*her3*)<sup>\*</sup>

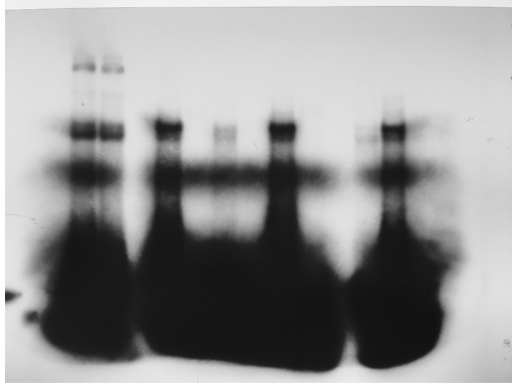

oligo 9

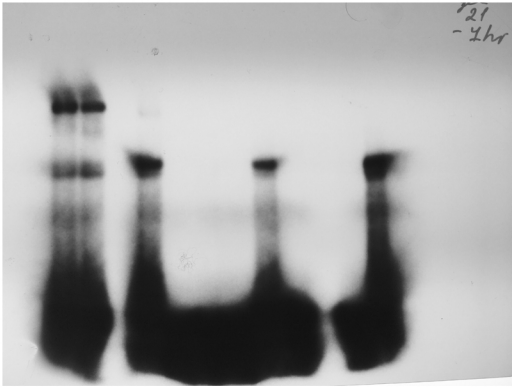

**b** 1.P+N+

oligo 1 (*myc*)

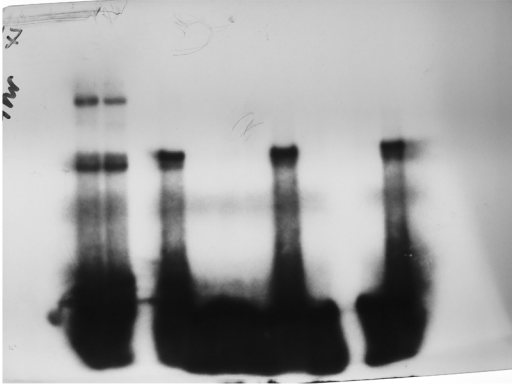

**c** 3.N+P-

oligo 5 (*marc3b*)<sup>\*\*</sup>

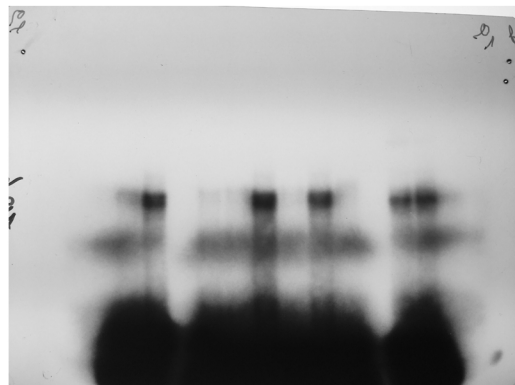

oligo 6

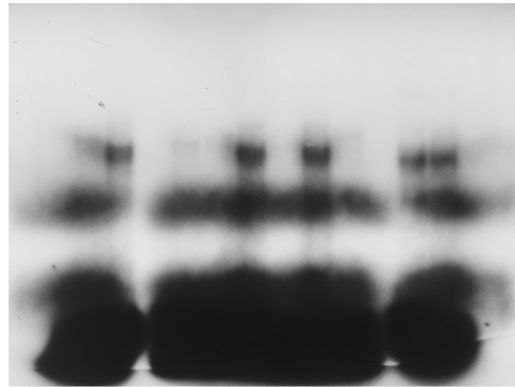

oligo 8

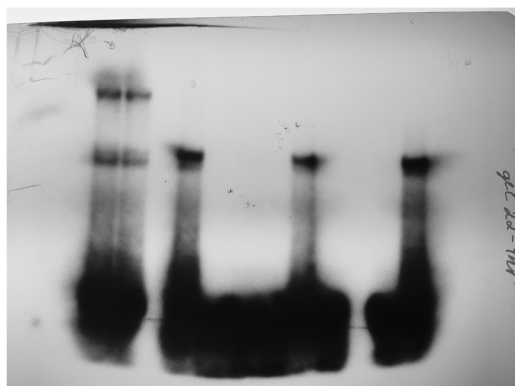

Supplement: Supplementary file 4 — Source Data [file 41467_2023_41507_MOESM4_ESM.zip › Source_data/Source_Data_4_uncropped_gels_Fig5.pdf]
